# *Mycobacterium marinum* Immune Evasion in Zebrafish

**DOI:** 10.3390/pathogens14090908

**Published:** 2025-09-10

**Authors:** Priyank Kumar, Joshua Cameron, Beatrice Saviola, Vishwanath Venketaraman

**Affiliations:** 1 College of Veterinary Medicine, Western University of Health Sciences, Pomona, CA 91766, USA; pkumar@westernu.edu; 2 Western University of Health Sciences, Pomona, CA 91766, USA; jcameron@westernu.edu (J.C.); bsaviola@westernu.edu (B.S.); 3 Department of Biomedical Sciences, College of Osteopathic Medicine of the Pacific, Western University of Health Sciences, Pomona, CA 91766, USA

**Keywords:** zebrafish, *Mycobacterium marinum*, mycobacteriosis, granuloma, nontuberculous mycobacteria, immune evasion, innate immune system, adaptive immune system, macrophages

## Abstract

Fish mycobacteriosis, a chronic progressive disease caused by nontuberculous mycobacteria (NTM), affects marine, brackish, and freshwater fish. *Mycobacterium marinum* (*M. marinum*), the most important of the NTM, infects fresh and marine water fish causing necrotizing granulomas and associated morbidity and mortality. *M. marinum* causes disease in zebrafish in a dose-dependent fashion. The *M. marinum*-induced disease in the zebrafish is associated with the development of necrotizing granulomas with abundant bacteria in the necrotic areas. Acute infection with high infectious doses of *M. marinum* infection in zebrafish was characterized by uncontrolled replication of the pathogen and death of all fish within 16 days, while chronic infections were marked by the formation of granulomas in different organs and longer survival in the range of 4–8 weeks. This review therefore synthesizes recent advances in our understanding of *M. marinum*’s infection of zebrafish, molecular pathogenesis, virulence mechanisms, and immune evasion strategies in zebrafish, while also highlighting the host immune effector responses and the virulence mechanisms of *M. marinum*.

## 1. Introduction

Piscine mycobacteriosis is a common disease of marine, brackish, and freshwater fish [1,2,3], infecting more than 200 species of freshwater and marine fish in a vast region extending from the subarctic zone to the tropical one [4]. Fish mycobacteriosis has become an important threat to the aquaculture/aquatic industry, coinciding with the rapid development of sturgeon aquaculture in China [5]. Mixed mycobacterial infections have a significant economic impact, especially in the aquaculture and fisheries industry, as infection can reduce the production, eventually affecting the trade [6,7,8].

Fish mycobacteriosis is characterized as a chronic progressive disease caused by nontuberculous mycobacteria (NTM), as described by Novotny et al. [9]. Among these NTM species, *M. marinum* stands out as particularly significant. This opportunistic pathogen colonizes both fresh and marine water environments, causing necrotizing granulomas similar to tuberculosis, resulting in significant morbidity and mortality in fish populations, according to Rallis and Koumantaki-Mathioudaki [8,10]. The clinical presentation includes multiple symptoms such as uncoordinated swimming, abdominal swelling, weight loss, skin ulceration, and the formation of white nodules as granulomas in vital organs including the liver, kidney, and spleen [11,12].

This review hopes to elucidate the current state of *M. marinum* research and seeks to connect the biology and immunology of zebrafish with genetic and molecular advances from *M. marinum* investigations. Connection of various aspects of host biology with microbial pathogenesis may improve the comprehensive understanding of *M. marinum*’s interaction with the host’s immune system. This review ties basic and molecular mycobacterial microbiology with pathogenesis in a zebrafish model, formulating a comprehensive review of the current state of research that may inform further investigations.

### 1.1. Zebrafish as a Model Organism

The adoption of zebrafish (*Danio rerio*) in mycobacterial research represents a paradigm shift in understanding host–pathogen dynamics. Over the last two decades, the zebrafish (*Danio rerio*) has emerged as a robust vertebrate model for studying host–pathogen interactions with *Mycobacterium marinum*. The optical transparency of embryos and larvae enables direct visualization of infection processes and granuloma development in real time, an advantage that is rarely achievable in mammalian systems [13,14]. Importantly, zebrafish larvae rely exclusively on innate immunity during their early life stages, allowing the contribution of innate defenses to be dissected independently of adaptive responses. In adult fish, the coexistence of innate and adaptive immunity provides a more complete view of host defense. Additional strengths of this system include high fecundity, rapid external development, and the ease of genetic manipulation. These attributes have facilitated medium-throughput drug screening, genetic analyses, and imaging-based investigations that advance our understanding of mycobacterial pathogenesis and inform translational tuberculosis research [15,16,17]. The genetic tractability of zebrafish enhances their utility through established mutagenesis techniques and transgenic capabilities. These combined attributes have established zebrafish as an invaluable platform for mycobacterial research.

### 1.2. Taxonomic Classification and Characteristics of M. marinum

*M. marinum* belongs to the genus *Mycobacterium*, family Mycobacteriaceae, order Actinomycetales. *M. marinum* is classified among the slowly growing mycobacteria (SGM), a distinction that separates it from rapidly growing mycobacterial species [18]. This classification is based on its in vitro growth characteristics, requiring more than seven days to form visible colonies on solid media, and is similar to other clinically significant mycobacteria like *M. tuberculosis* and *M. leprae*.

The bacterium is an acid-fast, rod-shaped, aerobic organism with a complex cell wall rich in mycolic acids, which contributes to its resistance to environmental stresses and antimicrobial compounds [19]. *M. marinum* is an opportunistic pathogen that primarily affects ectothermic animals, particularly fish in both freshwater and marine environments. Its optimal growth temperature range (25–32 °C) reflects its adaptation to the body temperature of fish and other cold-blooded hosts [20]. Although infections in humans are rare, it primarily infects the skin and fascia of the hands or lower limbs through abrasions or wounds, with lesions appearing after a prolonged incubation period [21]. Recent whole genome sequencing studies have revealed extensive genomic diversity among *M. marinum* strains, with distinct genetic clusters that may represent separate subspecies, highlighting the importance of strain selection in experimental studies and the potential for variable pathogenic outcomes across different *M. marinum* isolates [22].

### 1.3. Natural Habitat and Transmission of M. marinum

*M. marinum* naturally inhabits aquatic environments, including fresh, brackish, and saltwater ecosystems. The bacterium can persist in water and biofilms for extended periods, contributing to its environmental resilience and transmission potential [12,23]. The transmission dynamics of *M. marinum* between fish remain incompletely understood, though several routes have been identified.

The primary route of transmission appears to be oral, occurring through consumption of infected dead fish [24]. Additional transmission pathways include direct contact with affected fish skin or through the gills [11]. The bacterium can enter the host through abrasions or micro-injuries in the skin or mucous membranes, establishing infection in tissue macrophages [12].

Research has identified live feeds as potential vectors for *M. marinum* transmission in laboratory and aquaculture settings [24,25]. Peterson et al. demonstrated that *Paramecium caudatum* can act as a vector for mycobacteria, providing a useful animal model for evaluation of natural mycobacterial infections and demonstrating the possibility of mycobacterial transmission in zebrafish facilities via contaminated paramecia cultures [26]. Several studies have highlighted the association of free-living amoebae with mycobacteria, with various *mycobacterium* spp. Revealing the capacity of intra-amoebal survival inside vacuoles [27,28]. This finding has important implications for biosecurity practices in both research facilities and commercial aquaculture operations.

Significant outbreaks of *M. marinum* have been documented in various fish populations worldwide, including goldfish, striped bass, and hybrid striped bass in the United States [29,30], sturgeon in China [6], and captive-bred Australian lungfish [31,32]. These outbreaks highlight the economic and ecological significance of *M. marinum* in both wild and aquaculture settings.

### 1.4. Host Range and Specificity of M. marinum

*M. marinum* exhibits a broad host range, primarily affecting fish but also capable of infecting amphibians, reptiles, protozoans [21], and occasionally mammals, including humans [33]. In fish, the pathogen causes a tuberculosis-like disease [1] characterized by necrotizing granulomas that can affect multiple organs, leading to significant morbidity and mortality in both wild and captive populations [12]. *M. marinum* was first isolated in 1926 at the Philadelphia Aquarium from a fish suffering from diseases characterized by tubercles in various tissues, including the spleen and liver [1,34].

The bacterium’s impact extends beyond aquatic species, as noted by Rallis and Koumantaki-Mathioudaki (2007) [10], who identified *M. marinum* as one of the most common atypical mycobacteria causing opportunistic infections in humans [10]. Human infections typically present as cutaneous granulomas, often referred to as “fish tank granuloma” or “swimming pool granuloma,” affecting the extremities, particularly the hands and forearms [35]. These infections usually occur following direct contact with infected fish or contaminated aquarium water, especially in the presence of skin abrasions or wounds [33,34,36].

The ability of *M. marinum* to infect both fish and mammals makes it an excellent model for studying mycobacterial pathogenesis and host–pathogen interactions with potential translational implications for human tuberculosis research [37,38,39].

It is considered the most important fish pathogen, associated with multiple symptoms, e.g., uncoordinated swimming, abdominal swelling, loss of weight, skin ulceration, and white nodule formation as granuloma in the liver, kidney, and spleen in both fresh and marine water fish [11,12].

### 1.5. Genetic Similarities with M. tuberculosis and Other Atypical Mycobacteria

*M. marinum* is an acid-fast mycobacterial species that is closely related to other mycobacterial species within the *M. tuberculosis* complex, which includes *M. tuberculosis*, *M. africanum*, *M. bovis*, *M. microti*, and other species. In fact, *M. marinum* is the most closely related mycobacterial species outside the complex to these tuberculous species. As with other bacteria in the genus Mycobacterium, *M. marinum* has a genome which is rich in guanine and cytosine bases. *M. marinum* possesses a 6.6 Mbp genome with 5424 coding regions, 10 prophages, and a mercury resistance plasmid [21,40,41]. It has an extensive complement of PE and PPE proteins and numerous type VII secretion systems. *M. marinum* shares 3000 orthologs encoding proteins with 85% amino acid identity in common with *M. tuberculosis* orthologs [21]. *M. marinum*, however, possesses additional coding regions not present within the *M. tuberculosis* genome, and, in fact, contains the most coding regions of all pathogenic mycobacteria, including the *M. tuberculosis* complex and atypical non-tuberculous mycobacteria. This likely reflects *M. marinum*’s diverse lifestyle in the environment and within animal and protozoan hosts. *M. marinum* is also more distantly related to *M. avium* subspecies paratuberculosis and the non-pathogenic *M. smegmatis*. It has been theorized that *M. tuberculosis* and *M. marinum* evolved from a common ancestor that had inhabited environmental niches. In comparison, the genome of *M. tuberculosis* has become smaller over time, and as such, it has become a pathogen requiring the infection of human and primate hosts while losing its ability to colonize environmental niches. *M. marinum*, on the other hand, retained a larger genome and maintained a broader host range and conserved environmental reservoirs. Although *M. marinum* predominantly produces skin and soft tissue infections in humans, it can produce disease in animal models similar to disease caused by *M. tuberculosis* in humans and primates that results in necrotic caseous granuloma formation [21,42,43]. *M. marinum* is also closely related to *M. ulcerans*, which has a smaller genome but with 97% nucleotide identity with the *M. marinum* genome and has acquired a virulence plasmid that encodes an immunosuppressive polyketide toxin, which is unique to that species [21]. It is theorized that *M. ulcerans* evolved from *M. marinum* in part by acquisition of its virulence plasmid [21,41,42,44,45].

## 2. Host–Pathogen Interface in Zebrafish (Figure 1)

### 2.1. Zebrafish Immune System Overview

Zebrafish have been used for decades as a model organism to dissect the pathogenesis of immune response in *M. marinum*. The larval state results in a transparent organism in which researchers can observe *M. marinum* infection and immune response under microscopy as well as other methods [45,46]. In addition, larval zebrafish lack an adaptive immune response for approximately 3 weeks after fertilization, which allows for separate study of the innate immune response at this stage from the influences of adaptive immune responses. Using zebrafish as a model host, researchers have been able to dissect different stages of *M. marinum* infection as well as diverse immune responses [47].

Upon infection, *M. marinum* can be rapidly taken up by macrophages that can then migrate to epithelial tissue and can organize into granuloma structures. Within these granulomas, *M. marinum* initially disrupts and escapes the phagosome, entering the cytoplasm and consequently inducing apoptosis in these infected macrophages. Infecting *M. marinum* can also induce polymerization of actin within the cytoplasm and then can efficiently spread cell to cell between macrophages, thus spreading the mycobacterial infection, especially to susceptible macrophages that support *M. marinum* replication [48]. Thus, it has been shown that during initial infection by *M. marinum*, macrophages serve to allow initial replication and spread of *M. marinum* even as these innate immune cells strive to control the infection. Macrophages laden with *M. marinum* can also translocate to other zebrafish tissues to establish nascent granuloma structures. Ultimately, adaptive immunity develops at later stages, and *M. marinum* infection is somewhat controlled. Granulomas at this stage develop caseous necrotic centers, which can contain extracellular mycobacteria. However, ultimately, even development of adaptive immune response fails to control infection with *M. marinum* even at lower initial infecting bacterial numbers and allows for persistence of mycobacteria within zebrafish [16,49,50].

**Figure 1:**
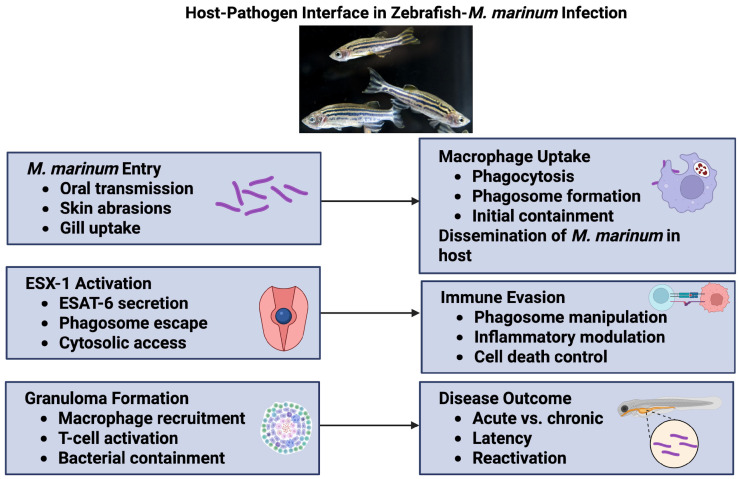
Schematic overview of the host–pathogen interface during *M. marinum* infection in zebrafish. The infection process begins with bacterial entry through various routes, followed by macrophage uptake, mycobacterial dissemination, and subsequent immune evasion mechanisms leading to different outcomes based on the immune status of the zebrafish. ESAT-1 activation and ESAT-6 secretion are important virulence mechanisms that allow *M. marinum* to escape the potentially growth-inhibitory environment of the subcellular phagosome of the zebrafish macrophage. ESX-1, EsxA, secreted by the ESAT-6 secretion system 1; ESAT-6, early secreted antigenic target 6 kDa. Created in BioRender 201. Kumar, P. (https://BioRender.com/r35ntbc accessed on 21 August 2025).

### 2.2. Cellular Components of Innate Immunity

Macrophages are an important part of the host’s immune system and serve in zebrafish as the initial defense against infection. It has been shown that *M. marinum*-laden macrophages can escape their resident granulomas and establish new granuloma structures in alternate locations within the zebrafish [50]. In humans during treatment of tuberculosis, as one site of infection within the lung resolves, other loci of infection develop, indicating that macrophages containing *M. tuberculosis* can migrate in the infected human. In addition, superinfecting *M. marinum* into zebrafish that have previously been infected and which have established granulomas results in superinfecting *M. marinum* trafficking within macrophages to occupy the previously established granuloma structures [13]. This mimics what has been seen in human disease caused by *M. tuberculosis*, where bacilli can superinfect and contribute to pathogenesis [41,51,52]. These results indicate that there are signals that draw macrophages laden with superinfecting *M. marinum* to occupy granuloma structures and migrate to their caseous centers.

### 2.3. Adaptive Immune Responses

Zebrafish develop a mature adaptive immune system as they exit the embryonic state after roughly four weeks after fertilization. Initially, innate immunity is important for the control of *M. marinum* bacterial growth; however, it can also contribute to the spread and dissemination of the mycobacteria within the host. Granulomas, the host immune structures that have a pivotal role in *M. marinum* pathogenesis, contain many cells of the adaptive immune system after the onset of adaptive immunity development. Adaptive immunity alters the granuloma structure to induce additional control of *M. marinum* replication, though these mycobacteria are never completely eliminated from the zebrafish organism [49,53,54,55]. Studies show that *rag1*-deficient animals as well as CD4 T cell-deficient animals have increased susceptibility to *M. tuberculosis* infections [49]. This has also been shown in adult zebrafish, which are *rag1* deficient and are more susceptible to *M. marinum* infection, as well as lacking B and T cells [37]. Though adaptive immunity does control infection to a degree, as observed in *M. tuberculosis* infection, much of the transmission occurs in immunocompetent individuals, pointing to the fact that adaptive immunity is not a guarantee of infection control. In addition, reinfection occurs frequently, indicating adaptive immunity is not effective to prevent this from occurring [49,56]. *M. marinum* has a similar infection profile in zebrafish, as the mycobacteria are not fully eliminated by the adaptive immune system and cannot induce protection from reinfection.

### 2.4. Pattern Recognition Receptors and Signaling Pathways

Toll-Like receptors (TLRs) on macrophages can interact with a variety of bacterial products, with TLR2 being important for interaction with products from *M. marinum*, which results in stimulation of cytokine production and migration of additional macrophages. Products which can bind TLR2 include *M. marinum* lipoproteins and lipoarabinomannan (LAM), which are important parts of the *M. marinum* cell wall. Interaction of mycobacterial products with TLR2 can activate signaling pathways in macrophages [21]. It has been discovered that phthiocerol dimycocerosates (PDIMs) are implicated in *M. marinum*’s ability to decrease association of *M. marinum*’s bacterial products by TLR2, resulting in subsequent evasion of bactericidal macrophages.

## 3. M. marinum Virulence Mechanisms (Figure 2)

### 3.1. Cell Wall Components and Their Role in Pathogenesis

*M. marinum* cell wall components contribute to the pathogenesis of this microorganism. And these components also interact with the zebrafish host. PDIMs are important in phagosomal membrane disruption and, additionally, the evasion of the host innate immune system [39,41,57]. The cyclopropanation of trehalose dimycolate, which is important in *M. tuberculosis* pathogenesis, has been indicated in vascular remodeling to produce granuloma angiogenesis [58]. Lipoproteins and LAM, important components of the cell wall, are also potent stimulators of the innate immune system through the host pattern recognition receptors [58].

**Figure 2:**
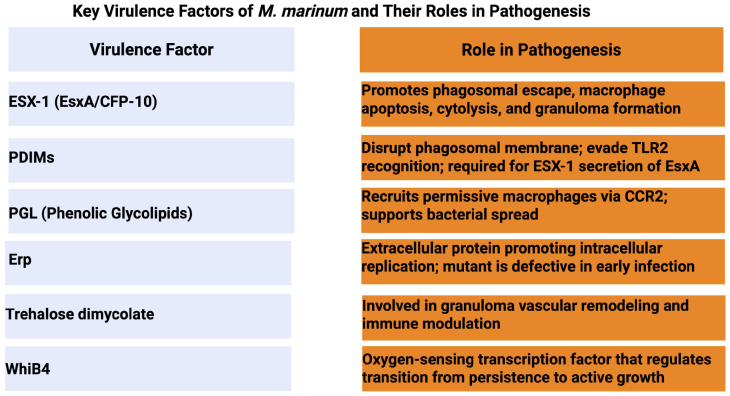
Key virulence factors of *M. marinum* and their roles in pathogenesis. *M. marinum* can evade immune action due to a variety of gene products, which can influence the outcome of infection in zebrafish. The above key virulence factors are listed with their associated roles in pathogenesis and modulation of the zebrafish immune system: ESX-1, EsxA, secreted by the ESAT-6 secretion system 1: EsxA, 6 kDa early secretory antigenic target, known as ESAT-6; CFP-10, 10 kDa culture filtrate protein; PDIMs, Phthiocerol dimycocerosates; Erp, exported repetitive protein; WhiB4, an oxygen-sensing transcriptional regulator in *M. marinum*; TLR2, Toll-like receptor 2; and CCR2, C-C chemokine receptor type 2. Created in BioRender. Kumar, P. (https://BioRender.com/p3wgb8v accessed on 21 August 2025).

### 3.2. Secretion Systems (Especially ESX-1)

*M. marinum* possesses nine type VII secretion systems. This recently discovered novel secretion system serves to export proteins from the mycobacterial cytoplasm to an extracellular location, which, during in vivo infection of macrophages, can cause mycobacterial products to enter phagosomes and the cytoplasm of the host cell. *M. marinum* has a large number of type VII secretion systems orthologs (nine) present in its genome, while *M. tuberculosis* has seven such orthologs. This large number possibly reflects the broader range of hosts infected by *M. marinum* as well as the utilization of environmental niches by this Mycobacterium, which may require more gene regulation modulation. *M. marium* ESX-1, a homolog of which is present in *M. tuberculosis*, has been shown to promote the export of ESAT-6 (Esx-A) and its chaperone CFP-10 from *M. marinum* [59]. It has been hypothesized that the ESAT-6, with the aid of CFP-10, can cause the lysis of the phagosomal membrane, ultimately resulting in apoptosis of the macrophage structure [60]. Subsequently it was determined that other secreted proteins may also be involved [61]. *M. marinum* can also stimulate hemolysis and cytolysis through ESX-1. In addition, a secreted protein, EspC, may be a secretion needle bridging the gap as to how these proteins exit the cytoplasm and traverse the cell wall and capsule [62].

### 3.3. Virulence Factors and Effector Proteins

The pathogenic mechanisms and virulence of *M. marinum* can vary significantly between different bacterial strains and host species. Ostland et al. demonstrated strain-specific differences in pathogenesis in zebrafish and hybrid striped bass, with ATTC 927 showing reduced pathogenicity compared to other isolates, while Broussard and Ennis revealed distinct virulence patterns between different fish hosts, specifically zebrafish versus medaka [24,29]. These findings underscore the importance of considering both strain selection and host species when interpreting *M. marinum* pathogenesis studies.

Type VII secretion systems have been important for exporting *M. marinum* bacterial products; the most important of these secretion systems is the ESX-1 system. ESAT-6 (Esx-A) secretion is facilitated by ESX-1 and is also implicated in lysis of the phagosomal membrane and mycobacterial escape from this subcellular compartment, as well as cell-to-cell spread.

PDIMs are located in the cell wall and are important for *M. marinum* virulence. It has been shown that PDIMs are required for full secretion activity of Esx-1 and the secretion of ESAT-6 (EsxA) and in promoting phagosomal disruption [57]. PDIMs may also contribute to immune system evasion through inhibition of mycobacterial cell products association with TLR2 [63].

Phenolic glycolipids (PGLs) promote the recruitment of macrophages that support the replication of *M. marinum* through the production of chemokine ligand 2 (ccl2) in initially infected bactericidal macrophages. This process stimulates the recruitment of permissive macrophages that can support the growth of mycobacteria and appears to occur via the zebrafish Chemokine Receptor 2 (CCR2) [64]. Additionally, infected bactericidal macrophages may fuse transiently with permissive macrophages, thus transferring *M. marinum* mycobacteria into new permissive cells to promote the infection process [65]. PGL expression has been linked to increased virulence in other mycobacterial species, including *M. tuberculosis*. In addition, mutants lacking PGL in *M. marinum* were attenuated in a zebrafish model [63].

Exported repetitive protein (Erp) is a mycobacterial extracellular protein with a modular structure, signal sequence for secretion, and PGLTS motif repeats. It appears to control colony morphology [66] and promotes the replication of *M. marinum* in macrophages [67]. A mutation in *erp* resulted in an *M. marinum* that is defective very early on in the infection process and is more permeable in vitro [67].

Trehalose dimycolate, another cell wall product, is also involved in virulence, and its modification in *M. marinum* is implicated in promotion of vascularization of granulomas within zebrafish [58].

### 3.4. Metabolic Adaptations During Infection

During infection, *M. marinum* undergoes extensive metabolic adjustments that allow persistence within the hostile environment of the host. Inside zebrafish granulomas, the bacterium encounters nutrient limitation, oxidative stress, hypoxia, and acidic pH. In response, stress-responsive regulons and genes involved in survival under starvation or redox imbalance are upregulated. These adaptations parallel those described for *M. tuberculosis*, suggesting conserved strategies among pathogenic mycobacteria [37]. Evidence from transcriptomic studies indicates that *M. marinum* can adopt a mixed physiological state, maintaining a basal level of replication while simultaneously activating persistence pathways. The transcription factor WhiB4 has been identified as an important regulator of this switch, serving as an oxygen sensor that facilitates reactivation from a quiescent state [21,48,50,68]. Such metabolic flexibility underscores the ability of *M. marinum* to establish long-term infections in zebrafish, making this model highly relevant for investigating persistence and reactivation in tuberculosis.

### 3.5. Immune Evasion Strategies

*M. marinum* infection in zebrafish (*Danio rerio*) serves as an invaluable model for understanding mycobacterial pathogenesis and host–pathogen interactions in aquatic organisms. Recent scientific advances have improved the understanding of *M. marinum*’s immune evasion strategies, highlighting the remarkable parallels between this pathogen’s virulence mechanisms and those of other mycobacterial species affecting marine life [39,69]. The zebrafish–*M. marinum* model has revealed sophisticated bacterial adaptations, including the ESX-1 secretion system’s role in phagosomal escape, manipulation of granuloma formation, and modulation of innate immune responses [39,61]. *M. marinum* exploits host macrophages as replication niches while simultaneously recruiting less bactericidal macrophages to facilitate bacterial growth and dissemination. The temporal dynamics of infection progression, from initial colonization to granuloma formation and bacterial persistence, provide crucial insights into therapeutic windows for intervention [39,61]. The zebrafish–*M. marinum* model’s optical transparency and genetic tractability have enabled real-time visualization of host–pathogen interactions and facilitated high-throughput drug screening, accelerating the discovery of compounds effective against mycobacterial infections in marine environments.

### 3.6. Manipulation of Phagosome Maturation

A key mechanism by which *M. marinum* evades host immunity is through the manipulation of phagosome maturation. After being engulfed by macrophages, *M. marinum* prevents the normal maturation of phagosomes into phagolysosomes, thereby avoiding exposure to antimicrobial compounds and creating a niche for survival and replication. Recently published studies demonstrated that ESX-1 affects host membrane integrity and induces type I interferon through separate genetic mechanisms. This study demonstrated that type I interferon production correlates with the release of mitochondrial and nuclear host DNA into the cytosol rather than bacterial DNA leakage [39,61,70]. This research provides significant insights into mycobacterial pathogenesis and suggests a revised model for ESX-1-mediated host interactions, which has implications for understanding tuberculosis pathogenesis.

SecA2, an ATPase present in mycobacteria that cause tuberculosis and leprosy, modulates the adaptive immunity to promote granuloma stability through induction of TNF-alpha [71]. Sullivan et al. (2012) demonstrated that the SecA2 system of mycobacteria subverts phagosome maturation to promote mycobacterial growth in macrophages [72]. By interfering with this critical host defense mechanism, *M. marinum* can establish persistent infection within host cells while evading immune clearance.

### 3.7. Interference with Inflammatory Responses

Reed et al. (2004) identified glycolipids in hypervirulent tuberculosis strains that inhibit the innate immune response [54]. Yu et al. in 2012 confirmed that *M. marinum* deficient in PDIMs/PGL were avirulent, indicating that both PDIMs and PGLs are necessary for virulence [63]. Additionally, a recent report by Xu et al. in 2024 confirmed that these bacteria can cleverly evade the host’s immune system by exploiting the transportation of trace metal elements [73]. *M. marinum* maintains the copper ion homeostasis by utilizing a sophisticated copper ion uptake system. Harjula et al. in 2020 shared valuable insights into genes important for defense against mycobacterial infections by demonstrating the utility of the zebrafish model for studying tuberculosis [52].

The ability to modulate inflammatory responses is crucial for establishing persistent infection while avoiding excessive immune activation that could lead to pathogen clearance. Recent research using the zebrafish model has provided insights into the molecular mechanisms underlying this delicate balance.

### 3.8. Modulation of Cell Death Pathways

*M. marinum* actively modulates host cell death pathways to favor its survival and dissemination. This microorganism both promotes and inhibits apoptosis at differing time points in the infection. Early in infection, apoptosis can be stimulated and is thought to increase *M. marinum* bacterial numbers that are available for additional macrophages to internalize. Later in infection, apoptosis can be inhibited. By inhibiting apoptosis and potentially promoting necrosis under certain conditions, the bacterium can manipulate host cell fate to create an environment conducive to its persistence.

Watkins et al. (2012) showed that M. marinum SecA2 modulates adaptive immunity to promote stable granulomas and induces TNF-alpha in vivo, highlighting the complex interplay between bacterial factors and host cell death pathways during infection [71].

### 3.9. Evasion of Adaptive Immunity

While much attention has focused on how *M. marinum* evades innate immune responses, strategies for evading adaptive immunity are equally important for establishing chronic infection. These include antigenic variation, inhibition of antigen presentation, and modulation of T-cell responses.

Recent research using the zebrafish model has demonstrated that zebrafish larval macrophages are polarized under challenged conditions [15,71]. The authors of this study elucidated that infected macrophages revealed downregulation of M2 markers (representing the anti-inflammatory activation state), while M1 markers (representing the pro-inflammatory activation state) were upregulated, with the strongest induction of a homolog of the human M1 marker CXCL11. A review of recently published literature suggests a potential target for mycobacterial immune evasion strategies [16,39,74]. By interfering with these pathways, *M. marinum* can potentially evade recognition by the adaptive immune system.

### 3.10. Granuloma Formation and Maintenance

The formation of granulomas, organized structures of immune cells surrounding infected macrophages, has traditionally been viewed as a host defense mechanism to contain infection. However, research using the zebrafish–*M. marinum* model has revealed that granulomas can actually facilitate bacterial persistence and dissemination under certain conditions.

Recent scientific advances in the zebrafish–*M. marinum* model have revealed sophisticated bacterial adaptations, including the ESX-1 secretion system’s role in phagosomal escape, manipulation of granuloma formation, and modulation of innate immune responses [39,75]. This research highlights how *M. marinum* exploits host macrophages as replication niches while simultaneously recruiting additional macrophages to facilitate bacterial dissemination.

## 4. Disease Progression Patterns

### 4.1. Acute Infection Dynamics

#### 4.1.1. Bacterial Replication Kinetics

*M. marinum* has an intermediate replication time compared to *M. tuberculosis* and *M. smegmatis*. Its generation time is between 4 and 6 h in vitro compared to over 20 h for *M. tuberculosis* and 2–3 h for *M. smegmatis*, although *M. marinum*’s generation time in vivo can be much longer than in vitro [21,37,75,76]. During infection, *M. marinum* is mostly found in granulomas and expresses genes generally found during the logarithmic phase of growth, indicating active growth during in vivo infection. Some mycobacterial granuloma-specific genes are upregulated as well, which are implicated in mycobacterial responses to stresses such as oxidative, acidic, and nutrient stress in vivo [77]. Other additional genes that also become active at certain points after initial infection that have been implicated in bacterial quiescence indicate a more complicated picture of replication, which may include persistence at later time points [77].

#### 4.1.2. Host Response Characteristics

Initially, macrophages arrive at the sites of infection and engulf invading *M. marinum*. These early-responding macrophages are manipulated by *M. marinum* and cannot efficiently control the mycobacterial infection. These macrophages responding to the infection can recruit additional macrophages and immune cells to stimulate nascent granuloma formation as well as additional mycobacterial replication. At times, macrophages, part of the early response and laden with *M. marinum*, travel to other locations within the zebrafish body to establish other loci of infection. The process of granuloma formation repeats itself with the establishment of granulomas at these distant locations. Eventually adaptive immunity increases, and macrophages become more competent to control and, in some cases, cause growth stasis of *M. marinum* in zebrafish [16,39].

#### 4.1.3. Mortality Patterns

*M. marinum* infection-induced mortality in zebrafish is dose dependent. Larger infectious doses of 8970 mycobacterial colony-forming units (cfu)/zebrafish caused a rapidly lethal infection within 2 weeks. With lower infectious doses of 5 cfu/zebrafish, 44% of the fish died of the infections, whereas an infectious dose of 60 cfu/zebrafish caused 83% mortality at 16 weeks. Generally, fish infected at higher cfu/zebrafish showed increased mortality with a variety of signs and symptoms. These included listless behavior of fish, where they remained at the bottom of the tank or on the surface constantly opening and closing their mouths to increase gas exchange [16,78].

### 4.2. Chronic Infection Features

#### 4.2.1. Granuloma Development Stages

Granuloma formation contributes to early expansion of bacteria. *M. marinum* utilizes the ESX-1/RD1 loci that are implicated in lysis of the phagosomal membranes, escape of the bacterium and its products into the cytoplasm of the host cell, and stimulation of apoptosis, which releases bacteria to be engulfed by recruited macrophages, which serve to facilitate additional mycobacterial replication. Thus, early in infection, the stimulation of apoptosis can encourage granuloma formation [53]. Overall, these zebrafish granulomas appear to have fewer macrophages present in these structures than human granulomas [16,37]. In addition, newly infected macrophages stimulate recruitment of additional macrophages that are less bactericidal and more mycobacterial replication-proficient macrophages through CCL2 ligand production and the CCR2 receptor on the recruited macrophages. As the disease progresses in zebrafish, macrophages can leave the initial granuloma structures and migrate to secondary sites where they initiate separate, distinct granulomas and additional foci of infection. This mimics what can happen in the human lung, where foci of infection can appear even as some other distant foci are healed due to antibiotic therapy. To control further mycobacterial growth, neutrophils arrive at sites where macrophages are infected with *M. marinum* and within granulomas where arriving neutrophils can contribute to infection control by engulfing, inactivating, and killing the mycobacteria. As the granulomas mature, the centers can become necrotic, as apoptosis is not a robust feature later in infection [79]. *M. marinum* can then be observed in the caseous centers of granulomas persisting extracellularly. Superinfecting *M. marinum* can also traffic to preexisting caseating granulomas [13]. While macrophages are important for control of *M. marinum* and within the context of granuloma formation, granulomas and macrophages, however, fail to eradicate *M. marinum* from the infected organism [13].

#### 4.2.2. Tissue-Specific Responses

Macrophages can depart from primary granulomas and can seed into a variety of tissues, possibly via a hematogenous route [53]. In addition, *M. marinum*, which is injected into zebrafish larvae via the caudal vein, can traffic to a variety of tissues. *M. marinum* can in fact traffic to the eye in larval zebrafish and has been shown to overcome the blood retinal barrier [53,80]. *M. marinum* can therefore be found within the eye tissue within macrophage aggregates, which are similar to those seen in other parts of the body. Hematogenous dissemination can result in prominent infection of *M. marinum* in tissues such as the spleen, kidney, and the liver [1,81]. Epithelial tissues are implicated as well by the induction of matrix metalloproteinase-9 (MMP9) due to ESAT-6 exposure. MMP9 recruits macrophages to the site of infection to enhance granuloma formation [82].

#### 4.2.3. Long-Term Survival Mechanisms

The outcome of *M. marinum* infection in zebrafish is strongly dose-dependent, ranging from rapidly progressive, lethal disease at high inocula to chronic infections that persist for weeks at lower doses [16]. In chronic models, granulomas become necrotic, and mycobacteria survive extracellularly within these lesions, recapitulating features of active human tuberculosis. Although bacterial numbers often stabilize at lower levels during long-term infection, *M. marinum* persists despite the presence of adaptive immune responses. Persistence is maintained by stress response pathways and specialized regulators such as WhiB4, which promote the transition between dormant and replicating states [16,21,68,83]. This capacity for prolonged survival highlights the zebrafish as a unique system for studying chronic mycobacterial infections, latency, and reactivation, and it provides an important comparative perspective alongside mammalian models of tuberculosis.

### 4.3. Host Immune Effector Responses

#### 4.3.1. Innate Immune Mechanisms

As the initial immune response to invading mycobacteria, innate immunity is of prime importance for control of mycobacterial infections and the outcome of these infections. Innate immunity is present continuously and may be more or less effective depending on the immune status of the zebrafish. As such, innate immunity serves as the first immune response encountered by invading mycobacteria, before the development of adaptive immune system responses to antigens roughly 10–14 days post antigen exposure. Macrophages, important cell types of the innate immune system, are the main controllers of mycobacterial replication and also, paradoxically, the dissemination mechanism for *M. marinum* within zebrafish. As macrophages are the preferred replication niche within the zebrafish organism, these host cells serve as a cell to support mycobacterial growth. Toll-like pattern recognition receptors present on macrophages recognize a variety of *M. marinum* products and can stimulate host cell signaling and cytokine production. Macrophages respond to invading mycobacteria by phagocytosing the invading pathogens and increasing reactive oxygen and nitrogen intermediates and decreasing pH within the phagosomes in an attempt to control mycobacterial replication. *M. marinum* responds by upregulating various gene products that combat the innate immune system.

#### 4.3.2. Cytokine and Chemokine Profiles

There are a number of cytokines which are important in the control of *M. marinum* replication. One of these cytokines is TNF-alpha, which appears to be important for infection control and progression of *M. tuberculosis* in humans, and likewise in zebrafish, TNF-alpha can promote the bactericidal effects of macrophages. Loss of TNF-alpha in zebrafish results in granulomas that are initially formed but fail to be maintained. Thus, in zebrafish, as in humans, TNF-alpha is important for macrophages to resist death and to maintain the granuloma structures, which likewise control *M. marinum* replication in vivo [84]. Upon *M. marinum* infection into the hindbrain ventricle, it is observed that macrophages migrate in response to the host cell expression of TNF-alpha and IL-1 beta [50]. TNF-alpha has been shown to promote necrosis via necroptosis of macrophages [85]. Necrosis is more efficient in its mycobacterial control, leading to less mycobacterial replication. This seems to occur later in the infectious process, while apoptosis is promoted earlier in infection with *M. marinum* and leads to greater survival and reuptake by arriving macrophages. Thus, promotion of necrosis and necroptosis by TNF-αlpha can aid in *M. marinum* infection control.

#### 4.3.3. Granuloma Structure and Function

The pathogenesis and granuloma formation patterns of *M. marinum* infection vary significantly across different fish host species, providing important context for interpreting zebrafish model results. Broussard and Ennis demonstrated that medaka (*Oryzias latipes*) exhibits greater resistance to *M. marinum* infection compared to zebrafish, with reduced mortality rates and a clear dose–response relationship regarding survival [24]. Similarly, earlier foundational studies using goldfish models revealed more chronic disease progression with lower mortality compared to zebrafish. Ruley et al. and Talaat et al. established these goldfish infection frameworks that preceded and informed the development of the zebrafish model, showing that host species selection significantly influences disease kinetics and outcomes [86,87]. These comparative studies highlight that zebrafish may represent a more susceptible host model, which should be considered when extrapolating findings to other fish species or interpreting pathogenic mechanisms.

Granuloma formation is the main mechanism of the host immune system to control an infection with *M. marinum*, though throughout the infectious process the mycobacteria are never completely eliminated. Granulomas in zebrafish appear to have overall fewer macrophages than human granulomas. Initially, apoptosis is stimulated by *M. marinum* using an ESX-1-dependent pathway to secrete ESAT-6 as well as other mycobacterial products. Apoptosis can be a factor in promoting bacterial release from infecting macrophages, after which bacteria are taken up by additional replication-permissive macrophages [53]. This process can promote replication of mycobacteria and expansion of the granuloma structure itself [53]. Mycobacteria that superinfect an already infected zebrafish can be taken up by macrophages and then translocate to already established granulomas [13]. Later, mycobacteria-containing macrophages can leave granuloma structures, enter other tissues, and establish new granulomas in diverse locations, thus serving as a mechanism for dissemination [50]. In addition, necrosis can allow bacteria to grow extracellularly in some circumstances. Thus, macrophages within the granuloma structure serve as a vehicle for spread within the zebrafish host.

#### 4.3.4. Adaptive Immune Response Development

The adaptive immune system develops roughly 4 weeks after fertilization in a zebrafish embryo. While initial innate immunity is important for control of *M. marinum* bacterial growth, innate immunity can also spread and disseminate the mycobacteria within the host via infected macrophages. Adaptive immunity can alter the process of granuloma formation and aid in control of *M. marinum* replication, though these mycobacteria may persist during the lifetime of the zebrafish [53]. Adaptive immunity develops 10–14 days after exposure to *M. marinum* antigens. This represents a critical response where the immune system can recognize specific antigens from *M. marinum* in addition to those recognized by pattern recognition receptors of the innate immune system. Adaptive immunity can change the outcome of an infection with *M. marinum* to allow for host immune control of invading mycobacteria.

## 5. Clinical Implications and Applications

Piscine mycobacteriosis, particularly that caused by *M. marinum*, poses a significant threat to fish aquaculture, even in strictly regulated research environments. The chronic and progressive nature of the infection can severely impact production yields in aquaculture settings; thus, limiting availability of embryos and sick fish can introduce compounding variables to research studies if undetected. There is also a risk to research staff. Findings from Mason et al. highlight the importance of a multi-faceted approach addressing both personnel and animal-related factors in controlling *M. marinum* outbreaks in zebrafish facilities. These suggestions include personnel wearing appropriate PPE and receiving adequate training as well as monitoring live feed contamination, providing appropriate animal transfer export disclosures, quarantining imported fish for 3–5 weeks, and embryo disinfection [14,88].

While animal models are crucial for understanding tuberculosis (TB) and discovering new drugs, most current models do not fully replicate human TB. For instance, mice, though cost-effective and widely used, exhibit distinct granuloma formation and increased resistance due to TB not being a natural mouse pathogen, yet remain the gold standard before human trials, whereas nonhuman primates, which do mimic human TB, are limited by cost, availability, ethics, and facility requirements [89]. The zebrafish model has emerged as a valuable addition, particularly in early research stages, due to its low cost, small size, high fertilization rates enabling statistical power, rapid and transparent development allowing real-time imaging of host–pathogen interactions, amenability to genetic manipulation, and ethical advantages for early larval stages [90,91]. For example, utilizing *M. marinum*, a close relative of TB that causes a similar but less severe disease allowing for BSL2 containment, zebrafish can be infected through various routes to study different aspects of TB pathogenesis and are particularly useful for in vivo drug screening, bridging the gap between in vitro assays and more complex mammalian models, thus potentially accelerating and economizing the drug development pipeline [92,93,94].

The utility of the zebrafish model also holds promise for vaccine development prospects. Niskanen et al. identified seven *M. marinum* genes upregulated during in vitro reactivation and further demonstrated that one of these antigens, MMAR_4110, could prevent the reactivation of latent mycobacterial infection in adult zebrafish, suggesting these genes as potential targets for TB vaccine and drug development [95]. Recently, Chen et al. used an mRNA tuberculosis vaccine delivered by lipid nanoparticles that demonstrated superior prophylactic and potent post-infection therapeutic activity against *M. marinum* in a zebrafish model of TB by activating DNA damage repair systems and autophagy. Their results highlight the potential of mRNA vaccines for TB [96]. The ability to dissect the host immune response, particularly the innate and adaptive arms, during different stages of infection provides a valuable platform for identifying key antigens and immune mechanisms that can be targeted by vaccination strategies. Understanding how zebrafish control or fail to control *M. marinum* infection can inform the design of effective vaccines for mycobacterial diseases.

Furthermore, the research utilizing the zebrafish model offers opportunities for biomarker identification. The ability to visualize host–pathogen interactions in real-time and to conduct genetic screens can help identify specific molecular signatures associated with different stages of infection, disease progression, and host immune responses. Myllymaki et al. demonstrated that dexamethasone can reactivate latent *M. marinum* infection in adult zebrafish, a process associated with lymphocyte depletion similar to humans, and identified RpfB and MMAR_4207 as protective post-exposure DNA vaccine candidates against reactivation [97]. Jia et al. identified 19 differentially expressed genes in a zebrafish model of latent *M. marinum* infection compared to chronic infection, highlighting Nos2b, TNF-alpha, Il1b, TNF-beta, TLR1, TLR2, and TLR4b as central immune-related genes potentially crucial for controlling the initiation of latent tuberculosis [56]. Results like these could be crucial for developing diagnostic tools for early detection of mycobacteriosis.

## 6. Future Research Directions

Despite significant advances in our understanding of *M. marinum* pathogenesis and host immune responses, several knowledge gaps related to the molecular mechanisms utilized by *M. Marinum* to evade host immunity, the role of host immunity to control the mycobacterial infection, and the influence of environmental factors like stress and microbiome composition on susceptibility to infection and disease progression remain to be addressed.

Addressing these knowledge gaps will require multidisciplinary approaches incorporating advanced imaging, single-cell analyses, and systems biology perspectives. Recent work by Dirks et al. exploring transcriptional responses in zebrafish larvae to both *M. marinum* and *M. tuberculosis* infection represents an important step toward understanding conserved host defense mechanisms against mycobacterial pathogens [75].

Recent technological advances in the fields of imaging, transcriptomics and proteomics, high-throughput drug screening, and CRISPR have revolutionized our ability to study host–pathogen interactions during mycobacterial infections.

The integration of these technologies with established zebrafish models promises to accelerate our understanding of mycobacterial pathogenesis and facilitate the development of innovative therapeutic strategies. For example, Habjan et al. utilized the zebrafish infection model to conduct an anti-tuberculosis compound screen, identifying a novel aspartyl-tRNA synthetase inhibitor with potential therapeutic applications [94].

Research on *M. marinum* pathogenesis has identified several potential therapeutic targets that could be exploited for developing novel antimycobacterial strategies [53,56,72,97,98,99,100].

Targeting these pathways could lead to more effective treatments for mycobacterial infections in both fish and potentially humans, given the significant similarities between *M. marinum* and *M. tuberculosis* pathogenesis. The zebrafish model provides an ideal platform for evaluating the efficacy and safety of such targeted approaches before advancing to more complex mammalian models.

The zebrafish–*M. marinum* model has proven invaluable for studying mycobacterial pathogenesis, but opportunities for further refinement and expansion exist. Improvements would further enhance the utility of the zebrafish–*M. marinum* model for fundamental research and translational applications.

## 7. Conclusions

Research using the zebrafish–*M. marinum* model has yielded several key insights into mycobacterial pathogenesis and host immune responses. It was recognized that invading *M. marinum* may disseminate within the zebrafish organism via infected macrophages. In fact, the innate immune system not only fails to efficiently control *M. marinum* replication but also actively disseminates and promotes the replication of *M. marinum* in zebrafish. The insights gained from zebrafish–*M. marinum* research has important implications for both clinical practice and future research. The zebrafish–*M. marinum* model’s significance in advancing our understanding of immune evasion mechanisms has practical applications in developing more effective treatments for mycobacterial diseases in aquatic organisms, which may include stimulation and promotion of appropriate responses of the innate immune system as well as promotion of adaptive immunity to key *M. marinum antigens*, which may have potential implications for broader mycobacterial research [75].

Advances in the study of infection transmission dynamics of the intracellular pathogen *M. marinum* demonstrated that live feed vectors like paramecia, brine shrimp, and rotifers can transmit mycobacterial infections to zebrafish [88] and underscored the importance of comprehensive biosecurity approaches in both research and aquaculture settings. These findings highlight the complex ecological dimensions of mycobacterial infections that must be considered in developing effective control strategies.

In conclusion, the zebrafish–*M. marinum* model has provided unprecedented insights into the intricate interactions between mycobacteria and their hosts, revealing both conserved and unique aspects of pathogenesis. Continued refinement and application of this model, in conjunction with emerging technologies and integrative approaches, promises to further enhance our understanding of mycobacterial infections and facilitate the development of more effective strategies for prevention, diagnosis, and treatment across diverse host species.
